# Surfing the Waves of the CMJ; Are There between-Sport Differences in the Waveform Data?

**DOI:** 10.3390/sports6040168

**Published:** 2018-12-08

**Authors:** James Parker, Lina E. Lundgren

**Affiliations:** 1 The Rydberg Laboratory for Applied Sciences, School of Business, Engineering and Science, Halmstad University, SE-30118 Halmstad, Sweden; lina.lundgren@hh.se; 2 Centre for Sport and Health Research, School of Health and Welfare, Halmstad University, SE-30118 Halmstad, Sweden; 3 Centre of Artificial Intelligence Research, School of Information Technology, Halmstad University, SE-30118 Halmstad, Sweden

**Keywords:** force-time, jump testing, kinetic assessment, principal component analysis, vertical jump

## Abstract

The ability to analyse countermovement jump (CMJ) waveform data using statistical methods, like principal component analysis, can provide additional information regarding the different phases of the CMJ, compared to jump height or peak power alone. The aim of this study was to investigate the between-sport force-time curve differences in the CMJ. Eighteen high level golfers (male = 10, female = 8) and eighteen high level surfers (male = 10, female = 8) performed three separate countermovement jumps on a force platform. Time series of data from the force platform was normalized to body weight and each repetition was then normalized to 0–100 percent. Principal component analyses (PCA) were performed on force waveforms and the first six PCs explained 35% of the variance in force parameters. The main features of the movement cycles were characterized by magnitude (PC1 and PC5), waveform (PC2 and PC4), and phase shift features (PC3). Surf athletes differ in their CMJ technique and show a greater negative centre of mass displacement when compared to golfers (PC1), although these differences are not necessarily associated with greater jump height. Principal component 5 demonstrated the largest correlation with jump height (R^2^ = 0.52). Further studies are recommended in this area, to reveal which features of the CMJ that relate to jumping performance, and sport specific adaptations.

## 1. Introduction

In sports with elements of lower body explosive movements, strength and power are often considered key factors for sports performance. In surfing, the athletes use their lower body strength and power to perform various manoeuvres and gain higher scores whilst wave riding. Although few studies have been carried out on surfing athletes there is some support to suggest that lower body power has an impact on the level of performance [1,2]. The impact of strength and power on golf performance is relatively well documented as cross-sectional studies have shown statistically significant relationships with strength [3], power [4,5,6], and clubhead speed (CHS). Training studies involving strength and power training have also shown improvement in both vertical orientated and CHS [7,8]. The countermovement jump (CMJ) is a common method to assess lower body neuromuscular function among surfers [1,2] and golfers [4,5,6,9,10] and has been associated with performance in both sports [10].

The CMJ has been proposed as an appealing test because it is quick to perform, non-fatiguing and requires minimal familiarization [11]. Furthermore, it is a common method among investigators monitoring lower-body power in both surfing [1,2] and golf [4,5,7,9,10]. However, most of these studies measure discrete variables such as peak force and CMJ height [4,5,7,9,10], and have not reported measurements, like the rate of force development or impulse, which can describe mechanisms associated with the ability to rapidly generate force. Recent studies in golf have attempted to investigate peak force and rate of force development in the isometric mid-thigh pull (IMPT) [6,12], and vertical jumps such as the CMJ. The IMTP is performed on a force platform where time and force data can be measured and recorded. Neither study [6,12] showed strong (r ≤ 0.6) relationship between the rate of force development (RFD) at 0–150 ms, 0–200 ms, or peak force and CHS. One explanation for these findings may be that the IMTP test may not utilise the stretch shortening cycle (SSC) and is an isometric exercise, which is more effective at measuring the ability to generate peak force. This is somewhat supported in the study by Wells et al. [6], who showed a strong (r ≥ 0.6) relationship between positive impulse in a number of different vertical jumps and CHS. There is a paucity of studies investigating time-series variables in movements which utilise the SSC, such as the CMJ, among surfers and golfers. Both Leary [12] and Wells [10] pre-selected key data points (e.g., RFD at 0–150 ms) to subsequently investigate. This approach can be limiting as pre-selection is dependent on a priori knowledge of the movement which may lead to abandoning potentially relevant data.

Understanding the individual movement characteristics (i.e., the shape of curves) requires statistical approaches, which analyse the entire movements represented by time-series data (e.g., waveforms or curves). Such approaches allow for biomechanical research to be exploratory prior to the generation of hypotheses and are less likely to discard relevant information [13]. Analyses of waveforms can give an insight into movement characteristics and have been applied to a range of areas in sports biomechanics including, rowing, running, cutting movements, and jumping [13,14,15,16]. In the case of the CMJ, a recent study [17] investigating the difference in waveform characteristics in the CMJ among junior and senior rugby players showed that power was greater at 94–96% of the movement for the senior players. Another study [15] investigating differences in the CMJ showed that movement characteristics immediately before and after peak power, and at the start of the eccentric phase, were greater in males compared to females. The previous two studies [15,17] gave an insight into movement phases that were significantly different between groups, but did not describe the movement characteristics specific to different movement strategies (also referred to as movement signatures) [18]. Principal component analysis (PCA) provides a means to accomplish this goal. PCA is a multivariate analysis technique that extracts orthogonal common sources of variation in a data set and assumes that a dataset can be reduced to a few common modes of variation [14]. The biomechanical features captured by PCA can be visually interpreted either via representative extremes method or single component reconstruction. Previous research in biomechanics has suggested that a combination of both methods may lead to the robust and clinically relevant interpretations of waveform data [19]. For example, previous research [14] has shown that PCA is able to identify potentially clinically important differences in frontal plane mechanics in knee cutting tasks.

To perform an analysis of a sequential task, such as the CMJ, previous studies have divided the movement into six different phases [11] (weighting, unweighing, braking, propulsion, flight, and landing), which can be derived from the force-time data. Understanding the movement characteristics for different groups of athletes throughout these phases, may provide greater insight for researchers, practitioners, and athletes to develop specific training strategies. PCA may provide a unique perspective on the role of movement patterns that characterise golfers and surfers. 

We expect our results to showcase the use of time-series analysis to provide useful insights into CMJ technique, by studying the variance in the sample using PCA and comparing the first few principal components between golfers and surfers. We hypothesize that the waveform analysis will show between-sport differences in the force-time characteristics.

## 2. Materials and Methods

### 2.1. Participants

Thirty-eight, high-level athletes aged 20 ± 2.8 years, with body mass 72.5 ± 9.9 kg, performed three separate countermovement jumps on a force platform. The participants were either high level golfers (male = 11, female = 8) or high level surfers (male = 11, female = 8). All participants were actively competitive, at a national or international level during the time of testing. This study was approved by the regional Swedish ethics committee and the Edith Cowan Human Ethics Committee (2016/12 and 8700, respectively) and all the participants gave written consent to participate in the study.

### 2.2. Design

This study was a cross-sectional, comparative study between golf and surfing athletes.

### 2.3. Data Collection

To perform the CMJ participants were required to stand in an upright position on a portable force plate with hands placed on hips. Participants performed three trials of the CMJ, with a self-selected depth and the instructions to jump as high as possible. Data were collected at two different locations, close to the normal training environment, and all CMJ trials were performed on one of two force plates, which have been used previously to assess CMJ [2,9]. All golfers performed the CMJ on a portable force platform (Force Platform, Ergotest Innovation, Porsgrunn, Norway) sampling at 200 Hz. This force plate consist of four uniaxial load cells with a combined capacity of 20,000 N, linearity of 0.2%, hysteresis 0.2% and a natural frequency of 760 Hz, and data were collected using the software provided by the manufacturer (MuscleLab 4020e, Professional Edition, Ergotest Innovation, Porsgrunn, Norway). All surfers performed the CMJ on a portable force plate (400 Series Performance Force Plate; Fitness Technology, Adelaide, Australia), sampling at 600 Hz. This force plate has four uniaxial load cells with a combined capacity of 10,000 N, hysteresis of 0.02% and a natural frequency of 1100 Hz. The plate was calibrated using 20 kg and 200 kg prior to every subject, to ensure linearity below 0.2%. Data were collected using the software package (Ballistic Measurement System (BMS); Version 2, Innervations, Perth, Australia), without any digital filter applied. To get the two force measurement systems to be comparable, the BMS data were down-sampled to 200 Hz. The reason for the down sampling of the surfer data was to have comparable data for both groups of athletes.

### 2.4. Data Analysis

The time series of force data was normalized to body weight on the magnitude axis, and to 0–100% of the movement cycle on the time axis, starting from the beginning of the unweighing phase, and ending at take-off from the force plate, using Mathematica 11.3 (Wolfram Research Inc., Champaign, IL, USA). All data was used as input for the variance matrices and each repetition represented a row in the matrix. PCA resulted in principal component-scores (PC) and time series loading vectors for each principal component, which were then used for further analysis. The PCA was performed using a custom script in Mathematica 11.3 (Wolfram Research Inc., Champaign, IL, USA), following the protocol of Deluzio and Astephen [20]. The loading vectors were determined by extracting the eigenvectors of the covariance matrix and to find the explained variance of each PC, the eigenvalues of the PC matrix were calculated. To determine jump height, the impulse-momentum method based on the force-time data was used [21]. 

### 2.5. Statistical Analysis

Each PC-score was analyzed with regards to normalized force and group (surfer or golfer), and qualitative interpretation of PCs was performed by examining the loading factors for each PC together with the observation of mean data curves of force plus and minus two standard deviations of each PC-reconstruction. The interpretation concerned magnitude, phase shift, and waveform features at a certain span in the movement cycle. Furthermore, a t-test was used to determine differences between surfers and golfers for each PC, respectively, and to examine if any of the PC-scores had a statistical relationship with CMJ jump height, a linear regression model was used to fit the data. Mathematica 11.3 (Wolfram Research Inc., Champaign, IL, USA) was used for all statistical analyses and *p*-values below 0.05 were considered significant. The magnitude of R^2^ value for the linear regressions were categorized as weak (<0.3), moderate (=0.3–0.5), or strong (>0.5) based on suggestion of Cohen [22]. Within-person reliability of the jump height was assessed using intraclass correlation.

## 3. Results

### 3.1. Principal Component Analysis

**Figure 1:**
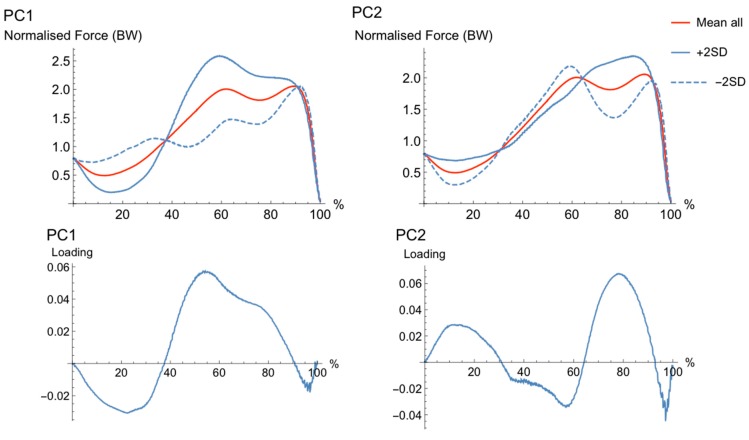
The pattern of variance, or loading vector, for PC1 and PC2. Mean waveform (solid, red), bounded by single PC reconstructed waveforms that represent only the variance due to the PC of interest for +2SD (solid, blue) and −2SD (dashed, blue) for “extreme” subjects. The loading vector for each PC is shown in the figure directly below the reconstructed waveforms for each PC.

**Figure 2:**
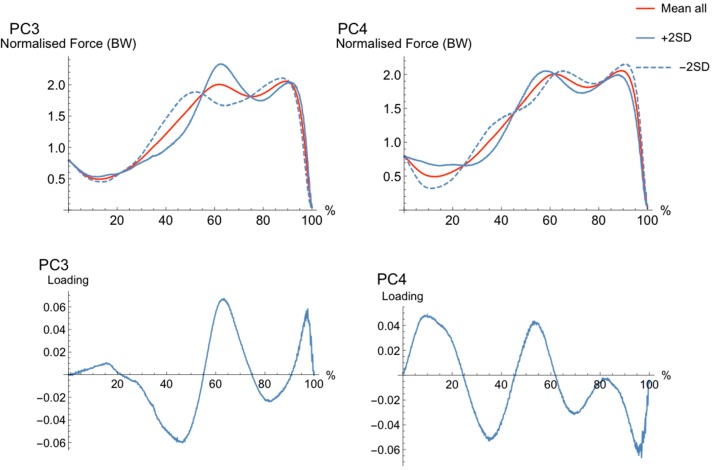
The pattern of variance, or loading vector, for PC3 and PC4. Mean waveform (solid, red), bounded by single PC reconstructed waveforms that represent only the variance due to the PC of interest for +2SD (solid, blue) and −2SD (dashed, blue) for “extreme” subjects. Loading vector for each PC is shown in the figure directly below the reconstructed waveforms for each PC.

**Figure 3:**
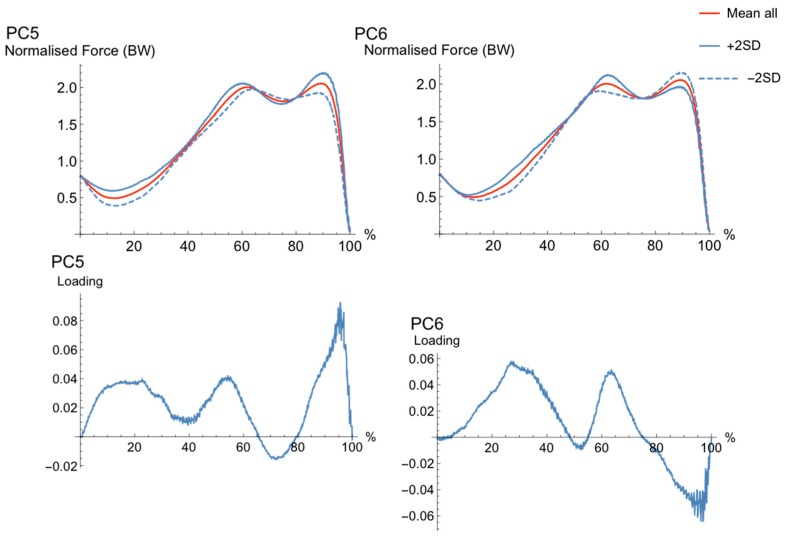
The pattern of variance, or loading vector, for PC5 and PC6. Mean waveform (solid, red), bounded by single PC reconstructed waveforms that represent only the variance due to the PC of interest for +2SD (solid, blue) and −2SD (dashed, blue) for “extreme” subjects. The loading vector for each PC is shown in the figure directly below the reconstructed waveforms for each PC.

The first six PCs explained 35% of the variance in force parameters. The main features of the movement cycles were characterized by magnitude (PC1 and PC5), waveform (PC2 and PC4), and phase shift features (PC3). Each PC and loading vectors can be seen in Figure 1, Figure 2 and Figure 3.

The waveforms for PC1, which were characterized by a difference in magnitude, (Figure 1) suggested that a high (+2SD) PC-score was associated with greater negative and positive acceleration (force) while a low (−2SD) PC-score was associated with lower negative and positive acceleration during the unweighing and braking phase of the CMJ movement. The first PC explained the most variance between 15–25% and 45–80% of the cycle, the high (+2SD) waveform had greater negative acceleration earlier (around 10–30% of the cycle) and greater positive acceleration during the middle of the cycle (45–80%) than the low (−2SD) waveform.

The waveforms for PC2, which were characterized by a waveform difference, (Figure 1) showed a difference in the shape of waveforms, and the high (+2SD) PC-score showed a continuous force peak compared to the low (−2SD) PC-score, which had a double peak in force development. The loading vector for PC2 explained most variance in waveform later in the movement cycle (70–80% of the cycle). The high (+2SD) waveform had a less negative acceleration earlier in the movement (5–25% of the cycle) and a slower rate of force development later in the movement (70–90% of the cycle). PC2 loading is greatest after the low (−2SD) waveform where the force data represent a U shape (70–85% of the cycle) prior to the second peak, closer to the end of the movement cycle.

The waveforms for PC3 (Figure 2) showed a phase shift in waveforms, where the high (+2SD) PC-score showed two force peaks later in the movement cycle, with greater magnitude in the first peak when compared to the low (−2SD) PC-score. The loading vector for PC2 explained most of its variance in the waveform during the early-to-mid (35–50%), mid (60–70%), and late (95%) movement cycle. The high (+2SD) waveform had less force development earlier in the movement (35–50%) a greater magnitude in force mid-movement (60–70%) and a second peak slightly later in the movement (95%). The low (−2SD) waveform showed an earlier first and second peak, along with a lower force development during early-to-mid-cycle (35–50%) and later (90%).

The waveforms for PC4, which were characterized by a waveform difference, (Figure 2) explains different rhythms in the movement cycle, where the high (+2SD) PC4 shows a disrupted unweighing phase, although it has a steeper force development in the braking phase, compared to those with the low PC-scores (−2SD). Furthermore, the low PC (−2SD) has a slightly higher and later second force peak (take-off peak force). The main loading for this PC is around 10%, 35%, 50%, and 90% of the movement cycle.

The waveforms of PC5, which were characterized by a difference in magnitude, (Figure 3) identified a difference in waveform magnitude, the high (+2SD) PC-score showed greater magnitude slightly later in the second peak in positive acceleration compared to the low (−2SD) PC-score. The loading vector for PC5 explained most variance in the waveform late (90–95%) in the movement cycle. The high (+2SD) waveform had greater rate and magnitude of force development late in the movement cycle (90–95%), and the low (−2SD) waveform showed less clear and smaller second peak in acceleration during the final part of the movement cycle (90–95%).

The waveforms for PC6 (Figure 3) had differences between high PC (+2SD) and low PC (−2SD) in the transition between unweighing and braking, where those with a high PC-score seem to have a slightly more accentuated unweighing and braking phases than those with a low PC-score. Furthermore, those with a high PC-score have a larger second than first peak, and the opposite for those with a low PC-score.

### 3.2. Comparison between Groups and Correlation with Jump Height

**Figure 4:**
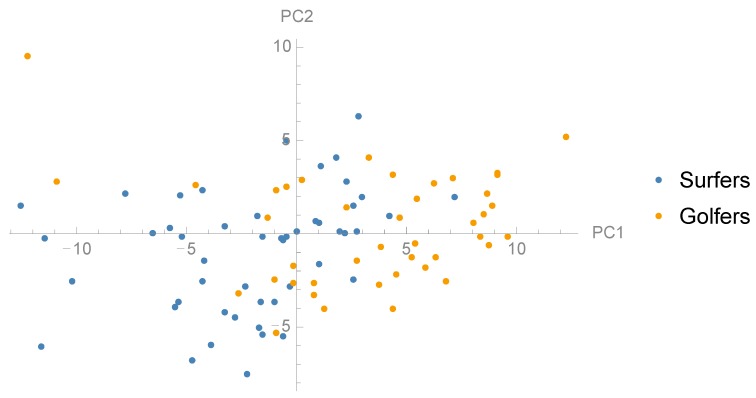
Scatter plot showing the distribution surfers and golfers in the dimensions PC1 and PC2.

**Figure 5:**
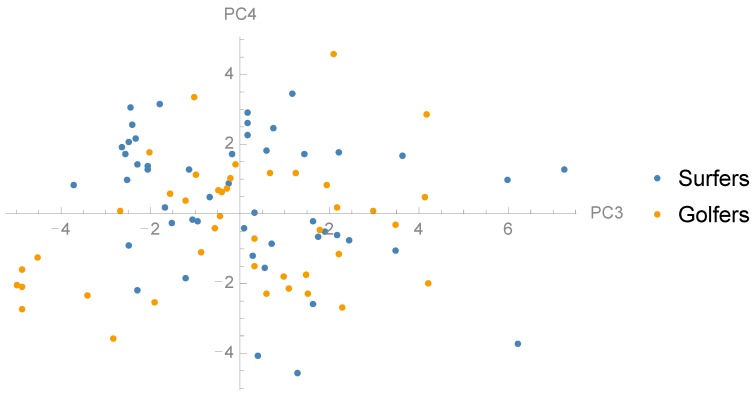
Scatter plot showing the distribution surfers and golfers in the dimensions PC3 and PC4.

**Figure 6:**
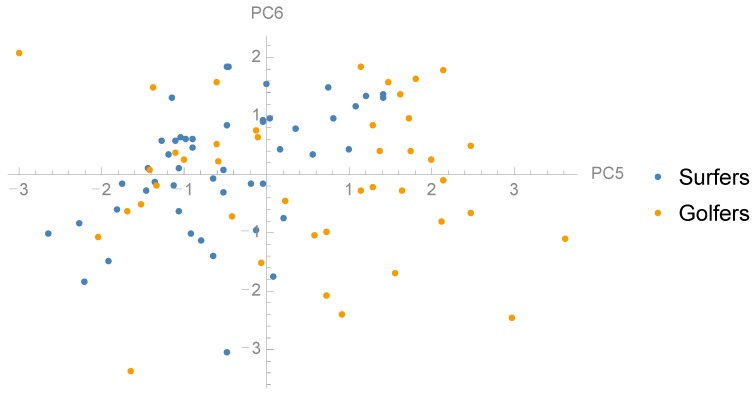
Scatter plot showing the distribution surfers and golfers in the dimensions PC5 and PC 6.

**Figure 7:**
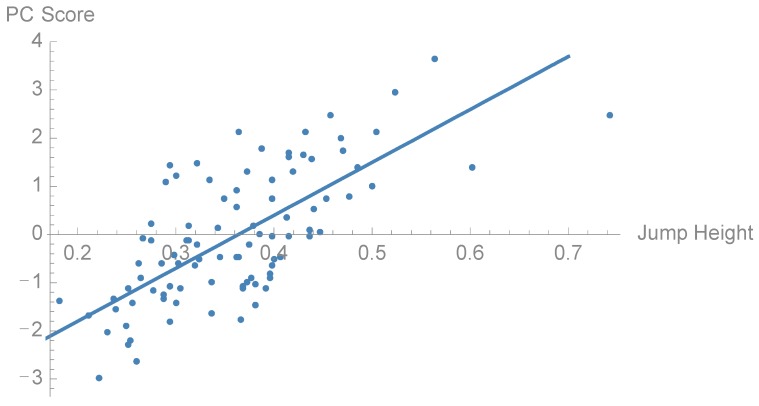
Scatter plot of the relationship between PC5 score and jump height (R^2^ = 0.52).

To examine whether there are any differences in the distribution of PCs between surf athletes and golf athletes we performed t-tests between the groups (Table 1). Golfers and surfers showed statistically significant different loading for PC1 (*p* = 0.001), PC2 (*p* = 0.014), PC4 (*p* = 0.027) and PC5 (*p* = 0.001) (Figure 4, Figure 5 and Figure 6). Furthermore, there was no significant difference between the groups in jump height (*p* = 0.536). The golf athletes had an average jump height of 0.37 (±0.11) m and the surf athletes 0.35 (±0.07) m. To examine the relationship between the PCs and CMJ jump height linear regression models were performed to investigate relationships between subject jump height and PC scores (Table 1). Four PCs, PC1, PC2, PC4, and PC5, showed a statistically significant relationship with jump height, in particular, PC5 showed a moderate to strong (R^2^ = 0.52) positive relationship to CMJ jump height (Figure 7). The within-person reliability of jump height was considered acceptable (ICC = 0.88).

## 4. Discussion

The purpose of this study was to examine waveform force-time characteristics during the CMJ among surf and golf athletes. It was hypothesized that PCA analysis would identify between-sport differences in CMJ force generation characteristics. The results in this study indicated that from the first six PCs, unique waveforms could be identified, of which four differed between groups. Only one of the PCs (PC5) had a moderate to strong correlation to CMJ height, which is the performance outcome of this specific test. PC5 explained about 4% of the total variance in this sample, which is a quite small part of the total. Nevertheless, it seems that the increase in jump height is mainly characterized by an increase in force both in the braking phase and peak force in the propulsion phase. This gives a high rate of force development in the propulsion phase of the jump, thus, a high peak velocity [23]. 

The unique feature types (magnitude, phase, and waveform) uncovered in this study are aligned with previous studies [14,19]. The first PC captured a magnitude feature mid-cycle (50–70%) and described that athletes who loaded high (+2SD) on PC1 had greater force production and a greater rate of force development throughout this phase in the cycle. The PC describes a phase directly after peak negative velocity through to a point just after peak force. This phase has been described as the braking phase, where the athlete decelerates the centre of mass (COM) [11], which coincides with the bottom of the countermovement. The greater negative acceleration exhibited by the higher waveform at maximum may be one explanation for the greater force production during the braking phase, and previous research has shown that the net impulse required during the braking phase is equal to the net impulse in the unweighing phase [23]. This phase has been described as countermovement-stretching or eccentric phase [23]. The *t*-test showed a between-sport difference in PC1 where golf athletes loaded higher than surf athletes (*p* = 0.001). Taken together, this suggests that golfers (who load high on PC1) apply more force into the ground during the braking phase than surf athletes. It may be assumed that golf athletes in comparison to surf athletes utilize a movement strategy that minimizes the braking time. Previous research has shown that higher negative COM velocity and, thus, greater braking forces are associated with greater CMJ performance [24,25], and support the movement strategy to maximize CMJ performance used predominantly by golf athletes. Interestingly the t-test tests did not reveal a relationship between PC1 and CMJ jump height, or any difference in jump height between the two groups of athletes. Previous research [26] has described how some athletes adopt a movement strategy that utilizes a greater negative COM displacement and a longer acceleration pathway to maximize CMJ performance. Greater negative COM displacement could describe the movement strategy used by surf athletes who have more time to develop force and thus could achieve similar net impulse and CMJ performance as the golf athletes. In our study we normalized time to percent of movement, thus the actual rate of force cannot be derived from these waveforms. 

PC2 also showed between-group differences, and accounted for about 10% of the explained variance. The surf athletes tended to have a lower PC2 score, indicating a greater dip between the first and second force peak of the braking and propulsion phase. This indicates a dip in the instantaneous impulse in the transition between phases, which, in turn, would require higher peak forces to achieve the same jump height as a more continuous waveform. Previous research [27] has described the waveforms represented by the high and low extremes as unimodal and bimodal shapes. It has been contended that the bimodal shape is indicative of more proficient jumpers. For example, studies have shown a development of bimodal shape after 12-weeks of jump training [24]. However a recent study [27] found no differences in jump height between the unimodal and bimodal shape among rugby union players, which is in line with our post hoc analysis that shows no relationship between PC2 and jump height. Kennedy and Drake [27] concluded that a bimodal shape is not an optimal movement pattern and simply reveals a more inefficient stretch-shortening cycle. This suggestion is in line with our results, however contrary to previous research suggesting bimodal shape may be a superior movement strategy in the CMJ [24]. The post hoc analysis did reveal a between-sport difference with surf athletes loading negatively on PC2, a low PC score (−2SD) described a bimodal waveform shape. This would suggest that the surf athletes, who loaded negatively on PC2 may benefit from training strategies that improve the stretch-shortening cycle which leads to an increase in positive net impulse during the propulsion phases. However, there is a paucity of research investigating changes in force-time waveforms and caution should be taken when applying training strategies to change waveform shape and movement characteristics in the CMJ.

Although PC4 also differed between groups, there seem to be little to discuss in relation to jump strategy. The increased variability in the low PC score (−2SD), which was more common among the golfers, may indicate that some of those athletes hesitated slightly in the braking phase and thereby created an extra peak during braking (Figure 1).

Athletes with a higher PC5 score were most of all golfers (*p* = 0.001). The loading vector for PC5 explained the most variance in the waveform late (90–95%) in the movement cycle, as well as around 10–15% and 55%. A biomechanical explanation for the relationship between PC5, jump height, and the greater force produced late in the cycle may be greater plantarflexion at the ankles among athletes exhibiting traits similar to the high PC score (+2SD). Ankle plantarflexion late in the CMJ movement will optimize positive COM displacement [11], and may describe movement characteristics which optimize jump height [26]. However, despite this PC being positively correlated to jump height, and being different between groups, no statistical significance in jump height was observed between the two groups. This may be due to different parts of the movement cycle having more or less influence in this PC, such as the less accentuated unweighing phase. Furthermore, there is only 4% explained variance from this PC on the waveform, which makes it a weak predictor for jump height as such. 

The limitations of this study are mainly related to the data quality and quantity, the two force platforms had mechanical differences in hysteresis (Ergotest Innovation = 0.2%, Fitness Technology = 0.03%) and sampling frequency (Ergotest Innovation = 200 Hz, Fitness Technology = 600 Hz). The difference in hysteresis, the loading and unloading of the force cells, and larger hysteresis of the Ergotest Innovation could lead to an overestimation of forces in the eccentric movement when compared to the Fitness Technology force platform. A higher sampling frequency ensures enhanced measurement precision, our CMJ data were recorded using two different sampling frequencies, 200 Hz and 600 Hz, and the Fitness Technology force data was then downsampled. Therefore, there is a possibility that fast events or force peaks have been missed in the recordings. The reason for the downsampling of the surfer data was to have comparable data for both groups of athletes. In this case we are looking at the general features of the waveform and not specific events or peak values. Therefore, we believe the low sampling frequency is justifiable although not ideal. Furthermore, the low number of athletes may account the little variance explained by the first six PCs. Ideally, around 80% of the variance should be explained within a few PCs, to provide a more useful result [20]. Furthermore, interpreting the biomechanical meaning of these parameters can be a subjective process. Biomechanical interpretations that are based on visual inspection of waveforms may be confounded when extreme waveforms express more than one biomechanical feature, which we are not able to filter out in this process. For future studies, a machine learning approach may be useful to explore in order to make the process less subjective. 

The CMJ has been associated with performance in both golf and surfing, however, the sport specific movements and training clearly differ between golf and surfing athletes. It is, therefore, appealing to hypothesize that athletes use movement strategies in the CMJ that reflect the sport they are involved in. The results of the current study provide useful information about how CMJ technique may differ between athletes in different sports, despite no significant difference in jump height between groups. The information may be used for training purposes, in case an athlete displays a force characteristic with less efficient features. For example, an athlete with a higher braking peak force than propulsive peak force may be advised to increase the push throughout take-off, or work on generating power through triple extension (hip, knee, and ankle).

## 5. Conclusions

Golf athletes and surf athletes differ in their CMJ technique, as shown by the different waveform feature captured by the PCA, however, these differences are not necessarily associated to jump height. Results from the PC1 show that surf athletes have a greater negative COM displacement and more time to develop force, thus achieve similar CMJ height as the golf athletes. PC2 showed that golf athletes exhibit a unimodal waveform which may indicate more reliance on the SSC to create positive impulse than surf athletes who exhibit a bimodal waveform. However, caution should be taken when applying training strategies to change waveform shape and movement characteristics in the CMJ as few studies have investigated such training interventions. Only one PC (PC5) showed a moderate to strong relationship to jump height. This may be associated with the high peak force feature during propulsion that was observed for those with higher PC5. Further studies are recommended in this area, to reveal which features of the CMJ that relate to jumping performance, and sport specific adaptations. 

## Figures and Tables

**Table 1:** Comparison of the six first principal components (PC) between surfing athletes and golf athletes. *p*-value < 0.05 denotes a significant difference between groups.

PC	Explained Variance (%)	R^2^ to Jump Height	Mean (±SD) Surfers	Mean (±SD) Golfers	*p*-Value
1	10.4	0.04	−1.99 (±4.23)	2.06 (±6.54)	0.001
2	10.4	0.01	−0.89 (±3.18)	0.81 (±3.43)	0.014
3	3.7	0.02	0.12 (±2.43)	0.01 (±2.49)	0.830
4	3.7	0.10 **	0.45 (±1.89)	−0.40 (±1.80)	0.027
5	3.7	0.52 **	−0.53 (±0.97)	0.57 (±1.61)	0.001
6	2.7	0.01	0.12 (±1.05)	−0.16 (±1.31)	0.247

** Significant correlation between PC and jump height, *p* < 0.01.
